# Cavaterm^TM^ *plus* treatment in high – risk surgical patients

**DOI:** 10.22088/cjim.12.3.336

**Published:** 2021-04

**Authors:** Zinatossadat Bouzari, Ebrahim Alijanpour, Shahla Yazdani, Azita Ghanbarpour, Ali Bijani, Tahereh Ashraf Ganjoei, Hemmat Gholinia

**Affiliations:** 1 Cellular and Molecular Biology Research Center, Health Research Institute, Babol University of Medical Sciences, Babol, Iran; 2 The Clinical Research Development Unite of Ayatollah Rouhani Hospital, Babol University of Medical Sciences, Babol, Iran; 3 Fertility and Infertility Research Health Center, Health Research Institute, Babol of University of Medical Science, Babol, Iran; 4 Social Determinants of Health Research Center, Health Research Institute, Babol University of Medical Sciences, Babol, Iran; 5 Department of Obstetrics and Gynecology, Preventative Gynecology Research Center (PGRC), Imam Hussein Medical Center, Shahid Beheshti University of Medical Sciences, Tehran, Iran; 6 Health Research Institute, Babol University of Medical Sciences, Babol, Iran

**Keywords:** Endometrial ablation, Menorrhagia, Amenorrhea, Recovery, Anesthesia, Obese women

## Abstract

**Background:**

The purpose of the study was to evaluate the effectiveness and safety of thermal balloon ablation in women with high anesthetic and surgical risk compared to invulnerable women according to the American Society of Anesthesia (ASA) physical status stratification.

**Methods:**

This report was based on a retrospective cohort study of women with heavy menstrual bleeding (HMB) who were eligible for treatment with Cavaterm^TM^ plus during 2012-2017. Women were classified as high-risk (HR) or low-risk (LR) cohorts based on ASA physical status stratification. The primary outcome includes amenorrhea in the twelfth months after the treatment. Risk adjustments were performed using regression models.

**Results:**

This research study consisted of 63 women with mean age 44.42±5.48. Mean of body mass index (BMI) in the HR cohort was higher than the LR cohort (31.48±6.22 vs 26.83± 3.51, P=0.005) and results were also similar considering the uterine length (mm) between HR and LR women (58.27±35.70 vs 30.92± 35.30, P=0.01). The primary outcome of treatment after a one-year follow-up in the two groups (HR and LR) was 31 (93.9%) and 15 (78.9%), respectively. After adjusting for known confounders including age, uterine length, parity, dysmenorrheal, the adjusted odds ratio was 0.94 (95% CI, 0.14– 2.5; P= 0.60).

**Conclusion:**

For women with high anesthetic and surgical risks derived from serious underlying co morbidities, endometrial ablation can provide a minimally invasive, safe, and effective therapy for heavy menstrual bleeding.

Heavy menstrual bleeding (HMB) is a common gynecological problem(1, 2) in women of reproductive and premenopausal age(1) that leads to anemia, fatigue, and reduces the quality of life(1, 3). Medical management is often the first-line treatment. When medical therapy fails, a hysterectomy is performed. Although hysterectomy is considered the “definitive” treatment for HMB, thermal balloon ablation (TBA) has become an increasingly popular treatment because of its minimal risk (4-6). The thermal balloon ablation includes four kinds of devices: ThermaChoice^®^, Menotreat ™, Cavaterm ™, and Thermablate ™. Cavaterm^TM ^is approved by the US Food and Drug Administration (FDA) for women with menorrhagia whose childbearing is complete (5, 7, 8). Cavaterm^TM^ (Wallsten Medical SA, Lausanne) “thermal balloon ablation is a second-generation, minimally invasive technique for the treatment of heavy menstrual bleeding (9) and there are many studies about the efficiency and safety of this technique (3, 10-14). Some patients with HMB also suffer from considerable co-morbidities that often preclude them from invasive surgical procedures(15).

Based on our knowledge, few studies have been published to evaluate the efficiency, safety, and patient satisfaction of this procedure (13). The aim of this study was to examine the outcome of treatment with Cavaterm^TM^ *plus *in women who are at high risk for surgery and anesthesia. The primary outcome was an amenorrhea rate at 12 months posttreatment with CavatermTM plus, whereas secondary outcomes were pain and patient satisfaction in the 3rd, 6th, and 12th months after treatment with CavatermTM.

## Methods

We performed a retrospective cohort study of women with HMB whose the pictorial blood assessment chart (PBAC) score was 100 or greater and were eligible for treatment with Cavaterm^TM^ plus (16), from December 20, 2012, through December 22, 2017. This study was approved before sampling by the Research Ethics Committee of Babol University of Medical Sciences (ethics code: MUBABOL.HRI.1391.12). 

Inclusion criteria consist of patients with prolonged uterine bleeding or heavy menstrual bleeding who were unresponsive to medical treatment, or all women with reported health problems who were considered as high –risk patients for hysterectomy. All women had undergone a sonography and it was used to rule out endometrial pathology and congenital anomaly. Women with uterine tumors (fibroids or polyps) were excluded. An endometrial biopsy was performed to assess endometrial cancer. Exclusion criteria included intra cavitary pathology (fibroids or polyps), a uterine cavity of less than four cm, an active urinary tract infection, pelvic infection ,the presence of coagulopathies, use of anti-coagulants desired to preserve fertility, history of surgery (myomectomy), endometrial ablation, and classical cesarean section.

Prior to preoperative care, patients were classified according to anesthetic risk. The American Society of Anesthesiologists’ (ASA) physical status classification stratified the study population into high-risk (HR) cohort and low-risk (LR) cohort, furthermore, the women were divided into low (ASA I and II) and high (ASA III and IV) anesthetic/surgical risk (17)

The Cavaterm endometrial thermal ablation was performed under general or regional anesthesia. The thermal balloon endometrial ablation depends on the transfer of heat from the heated liquid within a balloon that is inserted into the uterus (EEEE). The postoperative pain was assessed based on the visual analogue scale (VAS). The VAS is a straight line based on a scale of 0–10, where 0 stands for no pain and 10 for maximum pain. The pictorial blood assessment chart (PBAC) Scoring System was used to record the size of clots/flooding after the operation, the score of 100 and above indicated that the women had HMB and a score of zero defined ‘‘amenorrhea” (16).

 The primary outcome was the amenorrhea rate after 12 months from the treatment with CavatermTM plus. Failure rate was defined if there were no signs of amenorrhea after 12 months from the treatment in each group (HR and LR), whereas secondary outcomes were pain and patient satisfaction in the 3rd, 6th and 12th months after the treatment. Women completed health status checklists, including questions about the amenorrhea, reduction of menstrual flow, and heavy bleeding in the 3rd, 6th, and 12 months after the treatment. The patient’s satisfaction was also assessed in the 3rd, 6th, and 12 months after the surgery. The answer options include: excellent, good, medium, and poor. The collected data were coded and entered into the 18^th^ version of the SPSS program and was analyzed with t independent-test and chi-square tests. Risk adjustments were performed using regression models. Furthermore, p-values less than 0.05 were considered statistically significant.

## Results

Fifty-two women with HMB underwent balloon ablation in this study. Their mean age was 43/38 ± 5/91 years. The LR cohort included 19 women (15ASA I and 4 ASA II). The HR cohort consisted of 33 women (29 ASA III and 4 ASA IV). Patients in the HR cohort had higher BMIs than those in the LR cohort (P=0.005). Other characteristics were comparable between the groups shown in table 1. The HR cohort had more uterine length in women with heavy menstrual bleeding compared with those in the LR cohort (P= 0.01), but patients in the LR cohort had a higher score of bleeding than those in the HR cohort (P = 0.013) prior to the surgery (table 1). The mean duration of the anesthetic time was 13.35 min (S.D. = 1.84, range 12–16 min).

In all patients, after a one-year follow-up, the primary outcome of the treatment was 88.5% and six (11.5%) cases had a failure of treatment. All patients with a treatment failure were less than 45 years old and had not delivered. Also, their uterine length was less than 9 cm. The success rate of treatment after a one-year follow-up in the two groups (HR and LR) was 31 (93.9%) and 15 (78.9%), respectively. Nonetheless, endometrial ablation had the same efficacy in both the HR and LR cohorts during one-year (failure rates of 6.16% and 21.1% (P=017), respectively).

The outcome of Cavaterm^TM^ *plus* in high and low–risk surgical patients with heavy menstrual bleeding in the 3rd, 6th, and 12 months after surgery was shown in table 2. At 12 months, 17out of 33 (51.5 %) patients had amenorrhea in the HR cohort compared with 6 out of 19 (31.6%) in the LR cohort, with an unadjusted odds ratio of 0.55 (95% CI, 0.17–1.74; P=0.93). After adjusting for known confounders including age >45 years, uterine length ≤9 cm, parity >5, and dysmenorrheal, the adjusted odds ratio was 0.94 (95% CI, 0.14–2.5; P=0.60). The satisfaction of surgery in the 3rd, 6th, and 12 months after surgery in the HR cohort and LR cohort were not statistically significant (table 2). There were no complications including fluid overload, laceration of cervix, uterine rupture, and hematometra in both groups.

**Table 1:** Characteristics of the women in High and Low – Risk Surgical Patient with heavy menstrual bleeding undergoing endometrial ablation procedure (n=52)

**P**	**Patient group**		
**P-value**	**LR** **19 (36.5%)**	**HR** **33 (63.5 %)**	**Characteristic**
0.09	41.58±6.34	44.42±5.48	Age,yr (Mean±SD)
0.65	2.58±0.83	2.73±1.28	Parity (Mean±SD)
0.005	26.83±3.51	31.48±6.22	BMI, kg/m^2 ^(Mean±SD)
			Duration of menstruation, n (%)
0.09	2(10.5)	11(33.3)	4 -8 days
	17(89.5)	22(66.7)	>8 days
0.013	507.84±256.62	342.76±201.18	Score of bleeding (PBAC)
0.11	9(47.4)	23(69.7)	Preablation dysmenorrheal, n (%)
0.77	16(51.6)	15(48.4)	Previous cesarean delivery n (%),
0.89	11.10±1.66	11.15±1.29	Hemoglobin (g/dL), (Mean±SD)
0.01	30.92±35.30	58.27±35.70	Uterine length (uterine sounding), cm (Mean±SD)
			Anesthesia type, n (%)
0.69	16(84.2)	29(87.9)	Monitored anesthesia
	3(15.8)	4(12.1)	Regional
0.70	13.47±2.17	13.27±1.66	Operation time (minutes)

**Table 2:** Outcome of CavatermTM plus in High and Low – Risk Surgical Patient with heavy menstrual bleeding

**Outcome**	**HR**	**LR**	**P value**
pelvic pain/cramping at 1 hour(SD)	5.45±2.80	5.84±3.27	0.65
pelvic pain/cramping at 1 week (SD)	1.03±1.96	0.47±1.42	0.28
Amenorrhea rate at 3 months‘,n (%)	30(90.9)	12(63/2)	0.02
Amenorrhea rate at 6 months‘, n (%)	27(81.8)	11(57.9)	0.1
Amenorrhea rate at 12months‘, n (%)	17(51.5)	6(31.6)	0.24
Patient’ satisfaction rate at 3 months‘‘good’ to‘excellent’, n (%)	31(93.9)	17(89.5)	0.61
Patient’ satisfaction rate at 6 months‘‘good’ to‘excellent’, n (%)	29(87.9)	15(78.9)	0.44
Patient’ satisfaction rate at 12 months‘‘good’to‘excellent’, n (%)	31(93.9)	15(78.9)	0.17

## Discussion

Usually women with HMB who have had failed hormonal therapy apply for hysterectomy but hysterectomy is associated with the complications of major surgery (intraoperative or postoperative (18-21), especially in patients who were classified as HR cohort-based on the ASA physical status stratification. In this study, there was no significant difference in failure rate of treatment between the HR and the LR cohort (P= 0.17); it is similar to Mobolaji O et al. (2013). They reported after controlling for known confounders of treatment failure, failure rates remain unchanged(13). Other studies have compared endometrial ablation with hysterectomy for the treatment of AUB, finding that both have equally effective rates(15, 22) Endometrial ablation is a minimally invasive alternative to hysterectomy for abnormal uterine bleeding and its failure rate is low(23), considering that the incidence of obesity has increased dramatically over the last decade(24).

Anesthesiologists are increasingly being faced with treating obese patients(25). Obesity defined as the relationship between height and weight (weight [kg]/height2 [m2]), is measured by body mass index (BMI). The BMI is divided into five categories: <25 kg/m2 = normal, 25–30 kg/m2 =overweight, >30 kg/m2 = obese, >35 kg/m2 = morbid obesity, >55 kg/m2 = super morbid obesity(26). Obese women (BMI > 30) have a greater risk of complications than non-obese patients(27). Morbidity and mortality increases when the BMI is >30 kg/m2 and consequently, postoperative risks of hypoxemia and pulmonary complications are high in such women(28-30). Morbid obesity is associated with various pathophysiological changes, and it will affect the outcome of surgery and anesthesia. Planning for anesthesiology, various pathophysiological changes in morbid obesity should be considered(31). In this study, although patients in HR cohort had higher BMI compared with the LR cohort (P= 0.005), there were no complications with the anesthesia neither during the operation nor after the operation in both groups.

The shorter duration of endometrial ablation could explain the lower risk of surgery and anesthesia. The mean duration of the anesthesia was 13.35 min, in our study, also there was no difference in the duration of anesthesia between the two groups, but a hysterectomy involves a longer anesthesia overall. Prior reviews show that the mean duration of surgery was 56.4 minutes in an abdominal hysterectomy, whereas, it was 37.07 minutes in the vaginal(32). There was also blood loss during surgery, and the average hospital stay is longer with a hysterectomy(33). On the other hand, women with HMB also suffer from anemia, and preoperative anemia carries an increased risk of a longer hospital stay and increased postoperative morbidity and mortality regardless of the need for transfusion therapy (34-36). Age, obesity, duration of surgery, duration of hospital stay, and the mode of hysterectomy are known risk factors for postoperative patients (19, 20, 37-39). Consequently, patients may reject a hysterectomy as an initial treatment in HMB because it is invasive and requires time for recovery. In addition, women with menorrhagia are at a high risk because of bleeding disorders, morbid obesity, lung, and cardiac diseases, and other medical disorders, so, a thermal balloon endometrial ablation is safe and effective in treating abnormal uterine bleeding (AUB) in women who are stratified as HR according to the ASA physical status classification when other therapies are contraindicated or difficult to perform (40, 13).

## Conclusion

In the present study, although patients in the HR group had a BMI above 30, nevertheless, s endometrial ablation had comparable effectiveness both in the HR and LR cohorts. When women with HMB who had contraindications or that were difficult to perform a hysterectomy on , did not respond to medical therapy or other therapies, or reject a hysterectomy as an initial treatment, thermal balloon ablation is an effective and safe procedure.
